# The Association Between Caregiver Psychosocial Factors and Depressive Symptoms in People With Dementia: A Systematic Review and Meta‐Analysis

**DOI:** 10.1111/jan.17093

**Published:** 2025-06-25

**Authors:** Wenjing Ning, Shanshan Wang, Yudi Xu, Daphne Cheung

**Affiliations:** ^1^ School of Nursing The Hong Kong Polytechnic University Hong Kong SAR China; ^2^ Department of Neurology The First Affiliated Hospital of Zhengzhou University Zhengzhou Henan China; ^3^ School of Nursing and Midwifery, Faculty of Health Deakin University Melbourne Victoria Australia; ^4^ Centre for Quality and Patient Safety Research/Alfred Health Partnership, Institute for Health Transformation Deakin University Melbourne Victoria Australia

**Keywords:** caregivers, dementia, depression, meta‐analysis, psychology, systematic review

## Abstract

**Aims:**

To identify and evaluate the magnitude of the association between caregiver psychosocial factors and depressive symptoms among people with dementia.

**Design:**

Systematic review and meta‐analysis.

**Methods:**

A systematic review with meta‐analysis used a random‐effects model to estimate the effect size.

**Data Sources:**

Medline, PsycINFO, CINAHL, Scopus and Embase databases were searched for peer‐reviewed studies from inception to 25 November 2023.

**Results:**

The review included 88 articles, with 61 selected for meta‐analysis. Seven caregiver psychosocial factors were determined for the meta‐analysis: caregiver quality of life, distress, positive aspects of caregiving, depression, burden, quality of the relationship and anxiety.

**Conclusion:**

This study suggested that depressive symptoms in people with dementia were associated with caregiver quality of life, distress, burden, depression and positive aspects of caregiving.

**Implications for the Profession and/or Patient Care:**

Recognising the association between caregiver psychosocial factors and depressive symptoms in people with dementia has essential nursing implications. Adopting family‐centred care models and integrating respite care and psychological support for caregivers can help improve patient outcomes and overall dementia care.

**Impact:**

This study highlights the association between caregiver psychosocial factors and depressive symptoms in people with dementia. Caregiver distress, burden and depression were linked to increased depressive symptoms in people with dementia, while caregiver quality of life and positive aspects of caregiving were associated with depressive symptoms in people with dementia. These findings underscore the need for tailored interventions to enhance dyadic health.

**Reporting Method:**

This systematic review and meta‐analysis adhered to the Preferred Reporting Items for Systematic Reviews and Meta‐Analyses guidelines.

**Patient or Public Involvement:**

There was no patient or public contribution.

**Protocol Registration:**

This review was registered in PROSPERO (2024 CRD42024511383).

**Summary**

- Contributions to clinical community
  ○ This study highlights the importance of addressing caregiver psychosocial factors as a strategy to reduce depressive symptoms in people with dementia, ultimately improving patient outcomes.
  ○ Findings support the need for healthcare professionals to integrate caregiver mental health assessments and support programmes into dementia care, ensuring a more comprehensive intervention to dementia care.
  ○ The results emphasise the necessity for dyadic interventions in dementia care policies, advocating for tailored support programmes to enhance both caregiver psychosocial factors and depressive symptoms in people with dementia.
- What is already known about this topic?
  ○ Depressive symptoms are prevalent in people with dementia and negatively impact cognitive and functional abilities.
  ○ Caregivers play a crucial role in supporting people with dementia, and caregiver psychosocial factors are linked to depressive symptoms in people with dementia.
  ○ Some caregiver psychosocial factors, such as burden and distress, have been suggested to influence depressive symptoms in dementia patients, but findings have been inconsistent.
- What this paper adds
  ○ Caregiver distress and quality of life are moderately associated with increased depressive symptoms in people with dementia.
  ○ Caregiver depression, burden and positive aspects of caregiving are linked to fewer depressive symptoms in people with dementia.
  ○ There is no significant relationship between the dyadic relationship quality or caregiver anxiety and depressive symptoms in people with dementia.
  ○ The percentage of female caregivers moderates the association between caregiver distress and depressive symptoms in people with dementia.
- Implications for practice and policy
  ○ Interventions targeting caregiver psychosocial factors may help reduce depressive symptoms in people with dementia.
  ○ Healthcare professionals should assess and support caregivers' psychosocial factors as part of dementia care programmes.
  ○ Policymakers should integrate caregiver support strategies into dementia care policies to improve overall patient and caregiver outcomes.
- Impact statement
  ○ Addressing caregiver distress and quality of life can reduce depressive symptoms in people with dementia.
  ○ The findings support the integration of dyadic mental health assessments and targeted interventions into dementia care routines in clinical practice.
  ○ For education, it underscores the need to train healthcare professionals in recognising and managing caregiver‐related risks.
  ○ On a social and economic level, early intervention in caregiver psychosocial factors can reduce healthcare costs and promote sustainable dementia care at the community level.

## 1 Introduction

With the global population becoming older, the number of people living with dementia surpassed 55 million in 2020 and is projected to nearly double every two decades, reaching 139 million by 2050, with cases in low‐ and middle‐income countries (LMICs) rising from 60% to 71% (WHO 2021). This demographic shift highlights dementia care as a pressing international priority. The prevalence of depressive symptoms in people with dementia (PwD) has been reported as 33.2% in Alzheimer's disease (AD), 43.7% in Lewy body dementia (DLB), 60% in vascular dementia (VaD) and 33% in frontotemporal lobar degeneration (FTD) (Chakrabarty et al. 2015; Chiu et al. 2017; Cummings et al. 1987; Tetsuka 2021). Depressive symptoms may often present as the initial clinical indicator of early‐onset dementia and may persist throughout all stages of dementia without intervention (Huang et al. 2024). PwD who experienced depressive symptoms may have worsened memory impairment, further affecting their cognitive functions (Fang et al. 2021). An increasing amount of other evidence suggested that depressive symptoms in PwD had an impact on an increase in mortality (Wu et al. 2022). Beyond exacerbating cognitive decline and mortality risk, these symptoms impose significant global economic and social costs, estimated at $1.3 trillion annually (Alzheimer's Disease International 2023). Given the evidence that depressive symptoms were a modifiable risk factor for dementia (Watt et al. 2021), effective detection and management were critical to slowing progression and improving the outcome of the patient.

The provision and quality of dementia care were heavily influenced by family relationships and embedded social network dynamics (Wang, Liu, et al. 2024). Providing consistent help and support to PwD at home was often completed by informal caregivers (Huo et al. 2021). In high‐income countries, dementia care had higher costs, with greater reliance on formal care services and institutionalisation, whereas in LMICs, care expenses were lower but placed a significant burden on families, with the majority of individuals with dementia receiving long‐term, home‐based care from relatives (Alzheimer's Disease International 2018). Despite these differences, caregiver psychosocial factors universally influence patient outcomes, making this issue a linchpin for global dementia care strategies. This process of persistent care has profound implications for the psychosocial well‐being of caregivers and patients, emphasising the need to consider the binary relationship between patients and caregivers. The Family Systems Theory offered a valuable perspective here, suggesting that the psychosocial well‐being of caregivers and the depressive symptoms in PwD were interconnected, influencing each other within the family system (Watson 2012). Caregivers' emotional states were transmitted to PwD through close interpersonal interactions, potentially exacerbating depressive symptoms (Stall et al. 2019). As primary caregivers often spend the most time with PwD, their behaviour, emotional well‐being and coping strategies profoundly influence the behavioural‐psychological symptoms of dementia (BPSD) (Baharudin et al. 2019). Research highlights that caregivers play an essential role in shaping the daily experiences of PwD due to their sustained involvement in managing care routines and providing emotional support (Goh et al. 2024). Depressive symptoms in PwD were influenced by pathological (Esteban de Antonio et al. 2020), interpersonal (Monin et al. 2021) and environmental factors (Hendriks et al. 2024). However, unlike irreversible pathological contributors, caregiver psychosocial factors are modifiable through targeted interventions (Sun et al. 2022). Addressing caregiver stress and promoting effective coping strategies can foster a more positive caregiving environment, which may help alleviate depressive symptoms in PwD. Besides, environmental factors often require significant resources and long‐term interventions, making caregiver factors a more practical focus for immediate interventions.

This interconnectedness between caregiver psychosocial factors, including caregiver depression and anxiety (Ottoboni et al. 2019), caregiver burden (Ikanga et al. 2023), quality of life (QoL) (Nogueira et al. 2015), quality of relationships (Lewis and Riley 2021) and depressive symptoms in PwD illustrated a bidirectional relationship. Although there were numerous literature studies on the association between caregiver psychosocial factors and depressive symptoms in patients with dementia, the consistency of these findings was still unknown. The inconsistency in the research population, methods and sample size led to different reports. For caregiver burden, Wang et al. (2018) found that the correlation between caregiver burden and depressive symptoms in people with Alzheimer's disease was relatively high, at 0.749. However, there was a sharp contrast to Parrotta et al. (2020), which reported a much smaller effect size of 0.146, and Linton (2020) reported no relationship between them.

This review offered a comprehensive approach by incorporating diverse observational study designs, including cross‐sectional, cohort and case–control studies, to understand better the relationship between caregiver psychosocial factors and depressive symptoms in PwD. It uniquely focused on dyadic interactions, emphasising the interplay between caregiver factors and PwD depressive symptoms, which underscored the importance of addressing the needs of both parties in dementia care. Furthermore, the review tried to identify the key caregiver psychosocial factors most influential in contributing to depressive symptoms in PwD, providing valuable insights that could inform the prioritisation of interventions targeting these critical factors, thus enhancing the applicability and relevance of the findings.

## 2 The Review

### 2.1 Aims

This systematic review aimed to synthesise the caregiver psychosocial factors identified in the existing literature to estimate their correlation with depressive symptoms in patients with dementia. In addition, we hoped to evaluate the effect size of these caregiver psychosocial factors, providing more precise directions for future research and the development of targeted prevention plans. By identifying which caregiver psychosocial factors have the most substantial relationship with depressive symptoms in PwD, this study can provide crucial information on the priority order of interventions for informal caregivers, ultimately aimed at alleviating depressive symptoms in patients with dementia.

### 2.2 Design

We followed the Preferred Reporting Items for Systematic Reviews and Meta‐Analyses (PRISMA) (Liberati et al. 2009) throughout this review. This review has been registered at PROSPERO, and the registration number was CRD42024511383.

### 2.3 Search Methods

We have searched five databases, including Medline, PsycINFO, CINAHL, Scopus and Embase, which covered a range of health and social science disciplines. Besides, we retrieved the reference lists of the eligible literature. The cover article was published from inception to November 25, 2023. Keywords used include (carer* or caregiv* or “care giv*” or “care partner” or spous* or relative or famil* or couple or parent or child* or sibling) AND (Depression OR depressive OR depressed) AND (“Cognitive impairment” OR dement* OR Alzheimer*).

### 2.4 Inclusion/Exclusion Criteria

To determine the scope of the included literature, we established the following criteria for the included study based on the PEO (Population, Exposure of interest, Outcome) framework (Moola et al. 2020).

The subsequent inclusion criteria were implemented as follows: 1) The study included literature involving participants diagnosed with any type and severity of dementia and their caregivers. 2) The review examined caregiver psychosocial factors that might contribute to depressive symptoms in PwD. Caregiver psychosocial factors encompassed non‐biological, beyond attitudinal, behavioural and socio‐demographic elements, including support and networks (Tang et al. 2020). 3) The literature assessed the depressive symptoms in PwD. 4) Only literature published in English and accessible in full text online was considered. 5) Only studies published in peer‐reviewed journals were included in this review. The subsequent exclusion criteria were implemented: 1) the participants in the literature were restricted to exclude professional or paid caregivers. 2) conference papers that only had abstracts, letters, reviews, comments, editorials and books were excluded.

### 2.5 Search Outcome

The study underwent a rigorous screening process, with the first author initially screening titles and abstracts and the third author double‐checking. Relevant articles then proceeded to a full‐text review stage, where two independent reviewers assessed them based on eligiblity criteria in Rayyan (Khalil et al. 2022). Discrepancies were resolved by seeking consensus through consultation with a third senior team member. The result of the screening process was reported in full and presented in a PRISMA flow diagram (Page et al. 2021).

### 2.6 Quality Appraisal

The methodological quality of the included studies was assessed independently by two different researchers. Discrepancies were resolved through consensus. The Newcastle‐Ottawa Scale (NOS) was recommended to assess the quality of non‐randomised studies in meta‐analyses (Stang 2010). This tool has been used in evaluating case–control and longitudinal studies in previous systematic reviews (Coelho‐Júnior et al. 2021; Pereza et al. 2016). We used the NOS for quality assessment in this meta‐analysis instead of the GRADE approach because the NOS is well‐suited for evaluating observational studies, including cross‐sectional, cohort and case–control studies. While GRADE can also assess non‐randomised studies, it tends to start with lower certainty for such evidence (Schünemann et al. 2018), so we chose the NOS to address methodological concerns like bias and confounding factors more specifically.

In the case–control and longitudinal studies, NOS contains eight items: four scores for selection, two for comparability and three for outcome (Scores ≤ 4 points: low quality; 5–6 points: moderate quality; ≥ 7 points: high quality). NOS in cross‐sectional studies contains seven items: five scores for selection, two for comparability and three for outcome. In the previous literature (Tang et al. 2020), each study was classified as low (≤ 5 points) quality, moderate (6–7 points) or high (≥ 8 points).

### 2.7 Data Abstraction

A data extraction sheet tailored for the systematic review was used to extract data, including author, year, country, study design, diagnosis and sample size, setting, caregiver psychosocial factors, measurement for caregiver psychosocial factors, measurement for depressive symptoms in PwD and findings (What kind of relationship existed between caregiver psychosocial factors and depressive symptoms in PwD, their correlation coefficients, and whether there were other moderating or mediating factors between them). Two researchers screened the same 88 studies and independently extracted data from each study. Any arising disagreements were discussed with a third reviewer.

### 2.8 Data Synthesis

Meta‐analysis was conducted utilising Comprehensive Meta‐Analysis software version 3.0 (Biostat Inc., Engelwood, NJ, USA) (Brüggemann and Rajguru 2022), specifically in instances where a minimum of three studies reported the same caregiver psychosocial factors and employed the same study design. Results for the association between depressive symptoms in PwD and seven caregiver psychosocial factors (caregiver QoL: an individual's subjective evaluation and perception of their life (WHO 2012), caregiver distress: emotional, physical and mental strain from caregiving responsibilities (Lee et al. 2024), caregiver positive aspects of caregiving (PAC): caregivers encounter positive experiences while taking care of their loved ones (Yuan et al. 2023), caregiver depression: a loss of interest in people that once pleased you (Covinsky et al. 2003), caregiver burden: caregivers perceive that caregiving has had an adverse effect on their emotional, social, financial, physical and spiritual functioning (Liu et al. 2020), quality of relationship: individual's subjective evaluation and perception of positive and negative feelings about a relationship (Farooqi 2014), and caregiver anxiety (a highly distressing condition that is of particular importance in the context of caregiving (Del‐Pino‐Casado et al. 2021)) were pooled separately using random effects models. If the number of reports for a caregiver psychosocial factor in the included articles was less than three, we conducted a narrative synthesis for the other caregiver psychosocial factors. If the included articles reported multiple estimates, we used the adjusted estimates for a meta‐analysis. Publication bias was calculated using Egger's test to ensure methodological rigour, requiring a minimum of ten studies for reliability (Egger et al. 1997).

#### 2.8.1 Pooling of Effect Sizes

We used Pearson's correlation coefficient (*r*) in this meta‐analysis to calculate the effect size. Since Pearson's *r* was the most reported effect size in most of the included studies, it was chosen for its ease of understanding and interpretation (Rosenthal 1991). In addition, since fewer than three longitudinal studies reported the same caregiver psychosocial factors, this meta‐analysis does not include longitudinal studies. For studies reporting standard regression coefficients (*β*), *t*‐values or *p*‐values, these values were transformed into Pearson's correlation coefficients (*r*). The Comprehensive Meta‐Analysis software then transformed the correlation coefficients (*r*) to Fisher's *Z* for further meta‐analysis (Field 2001). For reporting of results, Fisher's *Z* was converted back to correlation coefficient (*r*) (Fisher 1992). Studies that only reported unstandardised regression coefficients, which could not be converted to standardised regression coefficients and had no alternative effect sizes, were excluded from the meta‐analysis.

#### 2.8.2 Study Heterogeneity and Meta‐Regression

The heterogeneity of effect estimation in the study was evaluated using Cochran's *Q*‐test and *I* ^2^ statistic (Higgins et al. 2003). The *I* ^2^ index was used to classify heterogeneity into low (<25%), medium (25%–50%) and high (> 75%) (Huedo‐Medina et al. 2006). If *I* ^2^ < 50% and *p* > 0.10, we used a fixed‐effects model. If *I* ^2^ ≥ 50% or *p* < 0.10 of the Cochran *Q*‐test, the exposure factors tested in the included articles were considered heterogeneous. The included studies were then further examined to explore the reasons for heterogeneity.

Due to missing data for certain predictor variables across different studies, a simple weighted least squares meta‐regression was conducted using a random‐effects model based on the method of moments approach. In meta‐regression, the analysis was conceptually like traditional regression at the individual level, but the unit of analysis is the study‐level effect estimate rather than individual participant scores (Xu and Doi 2020). The regression coefficients and their corresponding 95% confidence intervals (CIs) were calculated to evaluate the relationships between the outcomes and selected covariates. The meaning symbols (the red diamond) represented the pooled estimate or overall effect size along with its confidence interval. Results were considered statistically significant if the confidence intervals did not include zero. We planned to explore potential sources of heterogeneity by examining three key variables as moderators: caregiver age, caregiver gender (percentage of females) and patient age. Following the Cochrane Handbook guidelines, conducting meta‐regressions required a recommended minimum threshold of ten studies (Deeks et al. 2023). For studies where the confounder values were not reported in meta‐regression, an estimation was conducted by utilising the count‐weighted average of midpoints within confounder classes.

## 3 Results

### 3.1 Identification of Studies

**FIGURE 1:**
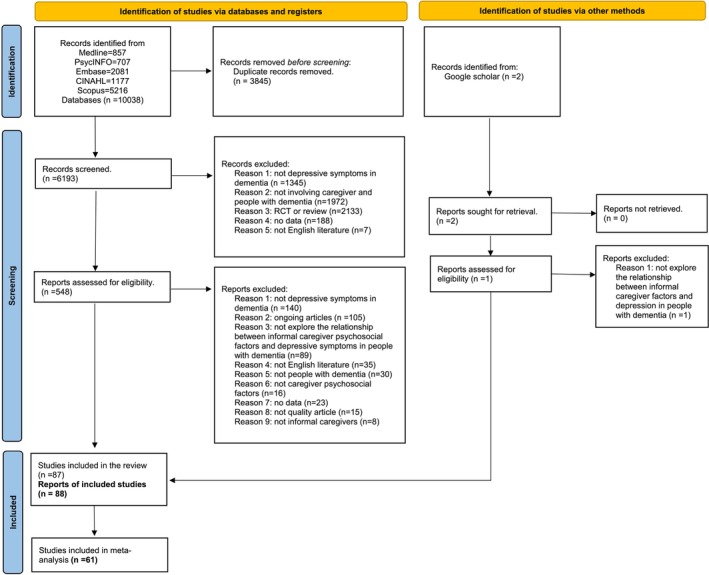
PRISMA flow diagram.

A total of 10,038 literature sources identified from Medline (*n* = 857), PsycINFO (*n* = 707), CINAHL (*n* = 2081), Scopus (*n* = 5216) and Embase (*n* = 2081). A hand search (*n* = 2) was conducted by reviewing the references of the related articles. After removing the duplicate papers, the remaining pool consisted of 6193 sources that required screening of their titles and abstracts. A total of 548 full‐text articles were retrieved and evaluated for eligibility. The final number of articles available for data synthesis was 88 (Figure 1).

### 3.2 Study Characteristics

Table 1 provided a summary of the characteristics of the included studies. All studies were published from 1997 to 2023. The majority of studies were conducted in the United States (*n* = 26, 29.5%). The entire 88 articles employed a quantitative methodology, among which 74 articles employed cross‐sectional study designs, while the remaining 11 utilised longitudinal designs, two cohort studies and another one utilised case–control design. The sample sizes of the included studies ranged from 14 to 1249 participants. Participants engaged in survey completion either through self‐completed questionnaires or via professional interviews conducted by trained personnel following questionnaire protocols. Different measurements of depressive symptoms in PwD were employed across the included studies. The most frequently utilised assessment instrument for evaluating depressive symptoms in PwD was the subscale of the Neuropsychiatric Inventory Questionnaire (*n* = 30) and the Cornell Scale for Depression in Dementia (*n* = 18). The most frequently (4/7) utilised assessment instrument for evaluating caregiver QoL was the Quality of Life in Alzheimer's Disease Scale. The Neuropsychiatric Inventory Questionnaire distress score was the most commonly (11/16) used assessment tool for measuring caregiver distress. A total of 19 studies assessed caregiver depression, with eight of them using the Center for Epidemiologic Studies‐Depression scale, three using the 9‐item Patient Health Questionnaire scores and two using the Beck Depression Inventory. Two‐thirds of the articles assessing PAC used the Positive Aspects of Caregiving Scale. Two out of six articles evaluating the quality of relationships used the Elder‐Caregiver‐Family Relationship Scale. Half of the articles assessing caregiver anxiety used the Zung Anxiety Scale. A total of 33 articles assessed caregiver burden, with 19 using the ZBI‐22, four using the ZBI‐12 and three using the CBI.

**TABLE 1:** Characteristics of the included studies in the review.

Author/year	Country	Study design	Diagnosis and sample size	Setting	Caregiver factors	Measurement for caregiver psychosocial factors	Measurement for depressive symptoms in PwD	Finding
Allegri et al. (2006)	Argentina	CSS	82 ad and their primary caregiver	Memory Clinic	Burden	ZBI	NPI‐subscale	There is no correlation between depressive symptoms in PwD and caregiver burden (rs = 0).
Ballard et al. (1997)	UK	CSS	47 married dementia (mild to moderate) and their spouses	Memory Clinic	continuing sexual relationship	Four additional questions	CDS	There was no significant association between depressive symptoms in PwD and a continuing sexual relationship (*r* = 0)
Bekhet and Garnier‐Villarreal (2020)	USA	CSS	100 dementia and their caregiver	Alzheimer's Association	Positive thinking; caregivers' burden (embarrassment/anger, patient's dependency and self‐criticism)	Positive Thinking Skills Scale (PTSS); ZBI‐22	Revised Memory and Behaviour Problems Checklist (RMBPC)	There was no association between depressive symptoms in PwD and caregiver positive thinking (*r* = 0), embarrassment/anger (*r* = 0), self‐criticism (*r* = 0) and patient dependency (*r* = 0) Caregiver's positive thinking did not play a moderation and mediation role between caregiver burden and depressive symptoms in PwD
Braun et al. (2010)	Switzerland and Germany	CSS	28 dementia (husband) and their wives	Clinic sample	Burden	The German version of the ZBI	CES‐D	There was no association between caregiver burden and depressive symptoms in PwD (rs = 0)
Brækhus et al. (1998)	Norway	CSS	92 mild dementia and their spouses	Memory Clinic	Stress	Caregiver Stress Scale	Behaviour and Mood Disturbance Scale (BMD)	There was a positive association between depressive symptoms in PwD and caregiver stress [social stress (*r* = 0.39) and depressive stress (*r* = 0.30)]
Bédard et al. (2005)	Canada	CSS	557 ad and caregivers	Memory Clinic	Burden	ZBI (role strain and personal strain)	GDS‐30	There is no association between depressive symptoms in dementia and role strain or personal strain (*r* = 0)
Brodaty and Luscombe (1998)	Australia	CSS	193 dementia and caregivers	Memory Clinic	Psychological morbidity	GHQ‐30	HDRS GDS‐30	There is a positive association between depression measurements in dementia [HDRS (*n* = 184,*r* = 0.21) and GDS‐30 (*n* = 124,*r* = 0.23)] and caregiver psychological morbidity
Bruvik et al. (2012)	Norway	CSS	230 dementia and caregivers	Community setting	QoL	QoL‐AD	CSDD (proxy‐rated)	Depressive symptom in PwD was negatively associated with caregiver QoL (*r* = −0.18)
Burgener and Dickerson‐Putman (1999)	USA	LS	84 early‐stage dementia and caregivers	Dementia diagnostic centre	Quality of the patient‐CG relationship Stress (Negative feelings towards patients, Social limitation, Personal distress)	ECFR Relatives' Stress Scale	CSDD	There was a positive association between the degree of depressive symptoms in dementia and the worse quality of relationships (*r* = 0.38) As for caregiver stress, depressive symptoms in PwD were not associated with personal distress but were associated with negative feelings towards patients (*r* = 0.42) and social limitations (*r* = 0.48)
Burgener and Twigg (2002)	USA	LS	73 dementia and their primary caregivers	Clinic sample	Quality of patient‐caregiver relationship	ECFR	CSDD (patient‐rated)	There is no association between depressive symptoms in dementia and the quality of the patient‐caregiver relationship (*r* = 0)
Cai et al. (2023)	Canada	LS	375 dementia and their caregivers	Memory clinic in rural area	Distress	NPI‐D	CES‐D	Baseline depressive symptomatology and its changes over one year in dementia patients were not associated with caregiver distress Baseline depressive symptomatology and its changes over one year in AD patients were not associated with caregiver distress
Caron et al. (1999)	USA	LS	72 dementia (mild to moderate) and their family caregiver	Community setting	Depressive symptoms Sense of mastery Boundary ambiguity (immobilisation: feeling of being trapped and overwhelmed in the caregiving role; closeout: emotional disengagement from the patient)	ZDS PMS Boundary Ambiguity Scale for Dementia	BEHAVE‐AD (subscale‐depression; proxy‐rated)	Initial caregiver depressive symptoms had a significant predictive relationship with the change in the patient's depressed mood over 12 months (partial r^2^ = 0.14) The patient's initial depressed mood was not predictive of the changes of caregiver's depressive symptoms at 12 months (*r* = 0) Initial caregiver immobilisation had a significant predictive relationship with the change in patient's depressed mood among 12 months (partial r^2^ = 0.07) and vice versa (partial r^2^ = 0.08) The effect of caregiver's perceived mastery or the caregiver closeout of patient on the change patient's depressed mood were not statistically significant and vice versa (*r* = 0)
Cheng, Ip, et al. (2013)	China (HK)	CSS	76 dementia and their family caregiver	Dementia service centres	Potentially harmful behaviours (psychological mistreatment and physical mistreatment) Burden Forgiveness	PHB scale ZBI‐12 Transgression‐Related Interpersonal Motivations Inventory	RMBPC‐subscale	Potentially harmful behaviours were not significantly associated with depressive symptoms in PwD (*r* = 0) Caregiver burden was correlated with depressive symptoms in PwD (*r* = 0.217) Caregiver forgiveness was correlated with depressive symptoms in PwD (r = −0.410)
Cheng, Lam, et al. (2013)	China (HK)	CSS	142 ad and caregivers	Mixed (memory clinics, outpatient clinics, day hospitals, day‐care centres or social service agencies, and another 62 were identified through another LS)	Burden Role overload Depression	ZBI‐22 Four‐item measure of role overload HDRS	RMBPC (subscale‐depression; proxy‐rated)	Caregiver burden was positively associated with depressive symptoms in PwD (*r* = 0.22) The role overload of caregiver was positively associated with depressive symptoms in PwD (*r* = 0.22) Caregiver depressive symptoms was positively associated with depressive symptoms in PwD (*r* = 0.23)
Conde‐Sala et al. (2009)	Spain	CSS	236 ad and caregivers	Memory Clinic	QoL	QoL‐AD	NPI‐subscale	Caregiver QoL was negatively associated with depressive symptoms in PwD (*r* = −0.318)
Dias et al. (2016)	Brazil	CSS	58 dementia (mild to moderate) and caregivers	Dementia outpatient clinic	Resilience	The Resilience Scale	CSDD	Caregivers' ability to positive adaption in the face of adverse life events was not correlated with depressive symptoms in PwD (*r* = 0)
Deeken et al. (2018)	Germany	CSS	80 dementia and caregivers	Memory clinics and private practices	Perceived stress	PSS	GDS‐15	Caregiver‐perceived stress was positively associated with depressive symptoms in PwD (*r* = 0.672)
Delfino et al. (2021)	Brazil	CSS	134 ad and caregiver	Geriatric clinic	Burden Depression	ZBI BDI	NPI‐subscale	Depressive symptom in PwD was associated with caregiver burden (rs = 0.35) and caregiver depressive symptoms (rs = 0.22)
Donaldson et al. (1998)	UK	CSS	100 ad and caregivers	Psychiatry outreach services	Subjective burden, Distress	GSS GHQ	CSDD	There is a positive association between depressive symptoms in dementia and caregiver subjective burden (*p* = 0.0063) and caregiver distress (*p* = 0.0022)
García‐Martín et al. (2023)	Spain	CSS	125 dementia and caregivers	Primary care setting	Burden	4‐item ZBI	NPI‐subscale	Depressive symptom in PwD was not associated with caregiver burden (*r* = 0)
Gellert et al. (2018)	Germany	LS	82 early‐stage dementia and caregivers	Mixed (memory clinics, specialty practices, nursing services and other social and medical institutions)	Dyadic coping	Dyadic Coping Inventory (DCI) subscales	GDS‐15	Caregiver's dyadic coping was not associated with depressive symptoms in dementia (*r* = 0)
Hallikainen et al. (2018)	Finland	LS	226 mild AD and family caregivers	Memory clinic	Psychological distress	12‐GHQ	NPI‐subscale	There was no relationship between caregiver distress and depressive symptoms in PwD (*r* = 0)
Hanzevacki et al. (2023)	Croatia	CSS	131 dementia and family caregivers	Health care centre	Burden	ZBI	NPI‐subscale	Depressive symptoms in PwD was predicted to caregiver burden (*p* = 0.008)
Harwood et al. (1998)	USA	CSS	653 ad and their caregivers (184 white Hispanic and 469 white non‐Hispanic)	Memory clinic	depression	CES‐D	Diagnosis by psychologists DSM‐IV through clinical interviews	There was no association between caregiver depressive symptoms and depressive symptoms in PwD (*r* = 0)
Hasegawa et al. (2014)	Japan	CSS	135 dementia and caregivers	Memory clinic	Depression	CES‐D	NPI‐subscale	There was a significant difference between patients in the depressive caregiver group compared with those in the non‐depressive caregiver group
Hiyoshi‐Taniguchi et al. (2018)	Japan	CSS	80 dementia and caregivers	Community setting	Distress Burnout	NPI‐D Pines' Burnout Index	NPI‐subscale	The severity of depressive symptom in PwD was associated with caregiver distress (*r* = 0.619) The severity of depressive symptom in PwD was associated with caregiver burnout (*r* = 0.267)
Huang et al. (2012)	China (Taiwan)	CSS	88 dementia and caregivers	Memory clinic	Distress	NPI‐D	NPI‐subscale	The severity of depressive symptoms in PwD was associated with caregiver distress (*r* = 0.370) The severity and frequency of depressive symptoms in PwD was associated with caregiver distress (*r* = 0.322) The frequency of depressive symptoms in PwD was not associated with caregiver distress (*r* = 0)
Huang et al. (2015)	China (Taiwan)	CSS	276 dementia and caregivers	memory clinic	Depression Kin relationship	CES‐D	GDS‐30	The degree of depressive symptoms in dementia was positively associated with the degree of caregiver depressive symptoms (*p* = 0.03) There was no difference between kin relationship and depressive symptoms in dementia
Ikanga et al. (2023)	Congo	CSS	30 dementia and caregivers	Community setting in rural	Burden	ZBI‐22	NPI‐subscale (proxy‐rated)	Depressive symptom s in dementia was predicted to caregiver burden (*p* = 0.0221)
Ilik et al. (2020)	Turkey	CSS	143 ad and caregivers	Outpatient neurology clinic	Burden Depression ISB	ZBI‐22 BDI ISB evaluated via interviews with caregivers	NPI‐subscale (patient‐rated)	Depressive symptom in PwAD was associated with caregiver burden (*p* = 0.000) and caregiver depressive symptoms (*p* = 0.002) PwAD who exhibit ISBs had higher levels of depressive symptoms than those who do not exhibit ISB (Z = −2.932; *p* = 0.003)
Iravani et al. (2022)	Iran	CSS	85 ad and caregivers	Hospital	Burden	CBI‐24	NPI‐subscale (proxy‐rated)	Depressive symptom in PwAD was associated with caregiver burden (*p* = 0.001)
Kai et al. (2022)	Japan	CSS	285 dementia and family caregivers	Psychiatric Hospital	Burden Depression	ZBI‐22 CES‐D	CSDD (Caregivers, physicians, nurses, pharmacists and psychosocial workers assessed together)	Depressive symptom in PwD was associated with caregiver depressive symptoms (*r* = 0.155) Depressive symptom in PwD was not associated with caregiver burden (*r* = 0) Depressive symptom in PwD was not predicted to caregiver depression and caregiver burden in multiple regression model
Kaufer et al. (1998)	USA	CSS	85 AD and their caregivers	Memory clinic	Distress	NPI‐D	NPI‐subscale	Caregiver distress was correlated with the frequency of depressive symptoms of PwD (*r* = 0.46) Caregiver distress was correlated with the total (frequency × severity) scores of depressive symptoms of PwD (*r* = 0.43) Caregiver distress was not correlated with the severity of depressive symptoms of PwD (*r* = 0)
Khoo et al. (2013)	Singapore	CSS	667 dementia and family caregivers	Memory clinic	Distress	NPI‐D	NPI‐subscale	Caregiver distress was correlated with the severity of depressive symptoms of PwD (*r* = 0.353)
Lethin et al. (2017)	European countries	Cohort study	1223 dementia and caregivers	Community setting	Burden	ZBI‐22	CSDD	Depressive symptom in PwD was predicted to caregiver burden (*p* = 0.024) Depressive symptom in PwD was associated with increasing caregiver burden (*p* = 0.005) More severe depressive symptoms in dementia among caregiver burden were significantly different compared with caregivers without burden at the baseline (*p* < 0.001) Caregivers who experienced an increase in the severity of depressive symptoms in dementia over time also had a statistically significant increase in caregiver burden compared that without increasing caregiver burden (*p* = 0.007)
Lewis and Riley (2021)	UK	CSS	35 dementia and spouse	Organisations providing dementia services	Communication ability Relationship continuity	Communicative Effectiveness Index Birmingham Relationship Continuity Measure	RMBPC‐subscale	Depressive symptom in dementia was not associated with communication ability and relationship continuity
Lima‐Silva et al. (2015)	Brazil	CSS	50 dementia (20 bvFTD and 30 ad) and caregivers	Outpatient clinics	Burden Distress	Short ZBI NPI‐D	CSDD	The degree of distress is partially correlated with the degree of depressive symptoms in dementia with the effect of caregiver burden eliminated in AD (*r* = 0.534), but this phenomenon does not occur in bvFTD (*r* = 0) The degree of caregiver burden is partially correlated with the degree of depressive symptoms in dementia with the effect of caregiver distress eliminated in AD (*r* = 0), but this phenomenon does not occur in bvFTD (*r* = 0)
Linton (2020)	USA	CSS	71 dementia (non‐Latina/o dyads = 42 Latina/o dyads = 29) and caregivers	Trauma Hospital	Burden Depression	CSI PHQ‐9	PHQ‐9	Depressive symptoms in dementia was positively correlated to caregiver depressive symptoms among non‐Latinas/o dyads (rs = 0.51) Depressive symptoms in dementia was not correlated to caregiver depressive symptoms among Latinas/o dyads and caregiver burden among Latinas/o dyads and Non‐Latinas/o dyads (*r* = 0)
Lou et al. (2015)	China	CSS	310 ad and caregivers	Neurological clinics	Burden Anxiety Depression	ZBI‐22 GAD‐7 PHQ‐9	NPI‐subscale	Depressive symptom in dementia was associated with caregiver burden (rs = 0.295), caregiver anxiety (rs = 0.429) and caregiver depressive symptoms (rs = 0.332)
Lu et al. (2022)	China	CSS	269 dementia and caregivers	A community and a nursing home	Distress	NPI‐D	NPI‐subscale	There was a strong positive correlation between depressive symptoms in dementia (depressive symptoms> = 4) and moderate‐to‐severe caregiver distress (depressive symptoms> = 3 (*p* < 0.0001)
Magai and Cohen (1998)	USA	CSS	168 dementia (mid to late stage) and caregivers	Alzheimer's Disease Center	Burden	BI‐22	BEHAVE‐AD (administered by a psychiatrist)	Depressive symptom in dementia was predicted to caregiver burden (*r* = 0.27)
Matsumoto et al. (2007)	Japan	CSS	67 dementia and caregivers	community setting	Distress	NPI‐D	NPI‐subscale	Caregiver distress was not correlated with the frequency of depressive symptoms of PwD (*r* = 0) Caregiver distress was not correlated with the total (frequency × severity) scores of depressive symptoms of PwD (*r* = 0) Caregiver distress was not correlated with the severity of depressive symptoms of PwD (*r* = 0)
Miller et al. (2019)	USA	CSS	42 dementia and caregivers	Adult inpatient acute care units	Depressive symptoms Care‐related Strain Relationship strain (caregiver self‐reported)	CES‐D Role Overload scale 5‐item Dyadic Strain subscale of the Dyadic Relationship Scale	CES‐D (patient‐rated)	There was no relationship between depressive symptoms in dementia and relationship strain evaluated by caregivers (*r* = 0), caregiver depressive symptoms (*r* = 0) and care‐related strain (*r* = 0) There was a positive association between relationship strain evaluated by PwD and depressive symptoms in PwD
Morgan et al. (2013)	USA	CSS	171 dementia and caregivers	Primary care and geriatrics clinics	Burden Quality of the patient‐CG relationship	Burden interview; Mutuality Scale	HAM‐D	There is a positive association between the degree of depressive symptoms in dementia and the degree of burden on the caregiver (*r* = 0.17) There is no relationship between the degree of depressive symptoms in dementia and the quality of the relationship of dyads (*r* = 0)
Neundorfer et al. (2001)	USA	LS	353 dementia and caregivers	community setting	Depression	CES‐D	BRSD‐subscale	More patient depressive symptoms predicted greater caregiver depressive symptoms at baseline (*r* = 0.16)
Nogales‐González et al. (2015)	Spain	CSS	222 dementia and caregiver	Social welfare and health centres	Self‐efficacy Distress	Revised Scale for Caregiving Self‐Efficacy‐subscale (Responding to Disruptive Patient Behaviours) RMBPC reaction‐subscale	RMBPC‐subscale	The frequency of depressive behaviours was associated with caregiver distress (*r* = 0.578) Self‐efficacy moderated the impact of depressive behaviours on caregiver distress for depressive behaviours
Nogueira et al. (2017)	Brazil	Case–control study	74 ad and spouse	Outpatient clinic	Sexual satisfaction	QSES	CSDD	There is no association between depressive symptoms in PwAD and caregiver's sexual satisfaction (*r* = 0)
Nogueira et al. (2015)	Brazil	CSS	54 dementia and spouse	Outpatient clinic	QoL	QoL‐AD	CSDD	There is a negative correlation between the degree of depressive symptoms in dementia and the level of QoL in Spouse‐caregivers (*r* = 0.97)
Ornstein et al. (2013)	USA	LS	160 probable AD and dementia with Lewy Bodies (DLB) and caregiver	Mixed (memory disorder centres or private physician offices	Depressive symptom	Brief Symptom Inventory‐subscale	Columbia University Scale for Psychopathology in Alzheimer's Disease‐subscale	The presence of depressive symptoms in the earliest stages of dementia was not independently associated with subsequent caregiver depressive symptoms when controlling for potential confounding variables at baseline (*r* = 0) Persistent depressive symptoms early in dementia were not associated with later caregiver depressive symptoms (*r* = 0)
Ornstein et al. (2013)	USA	CSS	160 probable AD and dementia with Lewy Bodies (DLB) and caregiver	Mixed (memory disorder centres or private physician offices	Depressive symptom	Brief Symptom Inventory‐subscale	Columbia University Scale for Psychopathology in Alzheimer's Disease‐subscale	After controlling all relevant confounders and symptom clusters, only patient depressive symptoms had a statistically significant impact on the likelihood of caregiver depressive symptoms (OR: 1.55, *p* < 0.01) Patient impact and caregiver‐perceived burden played a mediator role between patient depressive symptoms and caregiver depressive symptoms
Ottoboni et al. (2019)	Italy	CSS	355 ad and caregivers	Memory clinics	Burden Anxiety Depression Distress QoL	ZBI‐22 HADS NPI‐D EuroQuol	RMBPS‐subscale (proxy‐rated)	The frequency of depressive symptoms in dementia was associated with caregiver distress (*r* = 0.27) and caregiver burden (*r* = 0.26) There was no relationship between the frequency of depressive symptoms in dementia and caregiver anxiety (*r* = 0), caregiver depressive symptoms (*r* = 0) and caregiver QoL (*r* = 0)
Pang et al. (2002)	China (HK, Taiwan) USA	CSS	289 ad and caregivers	Mixed (outpatient clinic and research centre)	Distress Type of relationship	NPI‐D Spouse, close relatives and friends or other relatives	NPI‐subscale (proxy‐rated)	Caregivers in America had a higher sensitivity to patient depressive symptoms than caregivers in Taipei but did not differ from Hong Kong There was no difference between type of relationship and depressive symptom in dementia
Papastavrou et al. (2007)	Cyprus	CSS	172 ad and caregivers	Neurology clinics	Depression Burden	CES‐D BI‐22	MBPC‐subscale (proxy‐rated)	There was a positive association between depressive symptom in PwD and caregiver burden (*r* = 0.25) and caregiver depressive symptoms (*r* = 0.19)
Parrotta et al. (2020)	European countries	CSS	1223 dementia and caregivers	Community setting	Burden, Distress, Psychological well‐being, QoL	ZBI‐22 NPI‐Q GHQ‐12 EQ‐VAS	CSDD (proxy‐rated)	The degree of depressive symptoms in dementia was positively associated with the degree of burden (*p* = 0.001), distress (*p* = 0.002) and psychological well‐being (*p* < 0.001) The degree of depressive symptoms in dementia was negatively associated with the degree of health‐related QoL on the caregiver (*p* = 0.003)
Porta‐Etessam et al. (2011)	Spain	Cohort study	1249 moderate AD and caregivers	Nursing residence or hospital	Distress	NPI‐D	CSDD	Depressive symptoms in patients with moderate Alzheimer's Disease are significantly correlated with caregiver distress from baseline to 6 months
Pöysti et al. (2012)	Finland	CSS	335 dementia and spouse	NS	Burden	ZBI	CSDD	Cornell scores of the care recipient predicted caregivers' high burden (Zarit > 40 points) (*p* = 0.03)
Raggi et al. (2017)	Italy	CSS	90 ad (mild to moderate) and caregivers	Community setting	Burden	CBI	GDS‐15	In multiple regression, depressive symptoms in people with dementia significantly predicted caregiver burden (*r* = 0.408)
Regier et al. (2020)	USA	CSS	250 dementia and caregivers	community setting	Caregiver race Type of relationship Quality of relationship Burden Depression	Caucasian, African American, others Spouse, adult child, others QoL‐AD‐subscale ZBI‐12 PHQ‐9	NPI‐subscale	There was no relationship between caregiver race, relationship type and depressive symptoms in dementia Quality of relationship was negatively associated with depressive symptoms in dementia (*r* = −0.17) In the multiple linear regression model, depressive symptoms in dementia did not significantly predict caregiver depressive symptoms (*r* = 0) and caregiver burden (*r* = 0)
Robinson et al. (2001)	USA	CSS	30 dementia and caregivers	community setting	Impact from caregiving (value of caregiving, restrictions, health, Provoking nature, Economic cost)	Cost of Care Index (CCI)	RMBPC‐subscale	The frequency of depressive symptoms in dementia was associated with caregiver total impact (*r* = 0.45), caregiver restrictions (*r* = 0.49), emotional health (*r* = 0.39) and caregiver‐provoking nature (*r* = 0.39) There was no relationship between the value of caregiving (*r* = 0), economic cost (*r* = 0) and the frequency of depressive symptoms in dementia
Rosa et al. (2020)	Brazil	CSS	106 ad (mild to moderate) and family caregivers	Outpatient clinic	Resilience	The Resilience Scale	CSDD NPI‐subscale	In mild AD patients, the CSDD level or NPI‐depressive symptoms subscale was not associated with caregiver resilience (*r* = 0) In moderate AD patients, CSDD level was associated with caregiver resilience (*r* = 0.293), but NPI‐depressive symptoms subscale was not associated (*r* = 0)
Safavi et al. (2023)	UK	LS	61 dementia and caregivers	Dementia services	expressed emotion	Camberwell Family Interview (CFI)	GDS‐15	Caregiver EE status and critical comments were not related to depressive symptoms in dementia at baseline Caregiver EE status (df = 16.59) and critical comments (df = 8.76) were related to depressive symptoms in dementia at 6‐month follow‐up Caregiver EE status was predicted to depressive symptoms in dementia at the 6‐month follow (*r* = 0.702) Caregiver critical comments was predicted to depressive symptoms in dementia at 6‐month follow (*r* = 0.206)
Safavi et al. (2019)	UK	LS	61 dementia and caregivers	Dementia services	Distress	10‐item Clinical Outcomes in Routine Evaluation	GDS‐15	In multiple regression, caregiver distress did not significantly predict depressive symptoms in people with dementia at 6 months follow‐up (*r* = 0)
Sakar et al. (2019)	Iran	CSS	236 dementia and caregivers	outpatient clinics	elder abuse	Caregiver Abuse Screen (CASE)	GDS‐15	Depressive symptom in dementia was associated with abuse in elderly dementia patients (rs = 0.640)
Schumann et al. (2019)	Germany	CSS	100 AD and caregivers	Community settings or nursing homes	burden	ZBI‐22	NPI‐subscale	The depressive symptom was not related to caregiver burden (*r* = 0)
Shi and Scott (2023)	USA	CSS	108 ad and caregivers	community setting	caregivers' reactions (feel bothered or distressed) to depressive behaviours	RMBPC	RMBPC‐subscale	The frequency of depressive symptoms was predicted by Reactions to Depressive Behaviours (*r* = 0.703)
Shin et al. (2005)	USA	CSS	62 ad and caregivers	Memory Clinics	QoL	QoL‐AD	NPI‐subscale	There was no relationship between depressive symptoms in dementia and caregiver QoL (*r* = 0)
Simpson and Carter (2013)	USA	CSS	80 dementia and caregivers	Community setting	sleep quality	Pittsburgh Sleep Quality Index (PSQI)	NPI‐subscale	Depressive symptom in dementia was associated with subjective sleep quality (*r* = 0.26) but not associated with global sleep quality (*r* = 0)
Sousa et al. (2016)	Brazil and Spain	CSS	AD and their caregivers (Brazil (*n* = 128) and Spain (*n* = 146))	dementia units and memory clinics	Burden	ZBI‐22	NPI‐subscale	Depressive symptom in dementia was associated with caregiver burden in Spain (rs = 0.22) and in Brazil (rs = 0.28) Depressive symptoms in dementia were predicted to caregiver burden in male Spain subjects (*r* = 0.12), but not in female subjects (*r* = 0) Depressive symptoms in dementia were predicted to caregiver burden in male Brazil subjects (*r* = 0.53) and female subjects (*r* = 0.34)
Springate and Tremont (2014)	USA	CSS	206 dementia (mild to moderate) and caregiver (spousal or adult child)	Community setting	Burden (Impact on Caregiver's Life, Guilt and Frustration/Embarrassment)	ZBI‐22	RMBPC‐subscale	Depressive symptoms in dementia was associated with an impact on the caregiver's life (*r* = 0.24) and caregiver guilt (*r* = 0.22), but were not associated with frustration/embarrassment (*r* = 0)
Sutcliffe et al. (2017)	European countries	CSS	1223 dementia and caregivers	Community setting	Burden	ZBI	CSDD	The degree of depressive symptoms in dementia among high caregiver burden was not different with low caregiver burden
Tayeb et al. (2023)	Saudi Arabia	CSS	192 ad patients and caregivers	Community setting	Distress	NPI‐D	BEHAVE‐AD	The frequency of depressive symptoms in dementia was associated with caregiver total distress (*r* = 0.47)
Toda et al. (2018)	Japan	CSS	133 DLB and caregivers	Hospital	potentially harmful behaviour	Conflict Tactics Scale (CTS)	NPI‐subscale	Depressive symptoms in dementia did not play a significant role between those displaying potentially harmful behaviours (PHB) and those without such behaviours
Torrisi et al. (2017)	Italy	CSS	27 dementia (Alzheimer's disease, vascular dementia or frontotemporal dementia) and caregivers	Alzheimer's Care Unit	burden	CBI	NPI‐subscale	Depressive symptoms in dementia was predicted to the caregiver burden (*r* = 0.37)
Truzzi et al. (2012)	Brazil and Norway	CSS	145 dementia and caregivers	Outpatient clinic	burnout	MBI	NPI‐subscale	Depressive symptoms in dementia were not associated with caregiver burnout (*r* = 0)
Tsai et al. (2022)	China (Taiwan)	CSS	509 dementia and caregivers	Hospital	Burden	ZBI‐22	NPI‐subscale	Depressive symptoms in dementia were not predicted to caregiver burden (*r* = 0)
Victoroff et al. (1998)	USA	CSS	35 dementia and spouse	Outpatient dementia clinics	Burden Depression	ZBI, ZDS	CSDD	There was no significant association between patient depressive symptoms and either caregiver burden or depressive symptoms
Wagner et al. (1997)	USA	CSS	57 AD and caregivers	Outpatient dementia clinics	expressed emotion	Five‐minute speech sample (FMSS)	HDRS CES‐D	The mean and SD of depressive symptoms in dementia were not difference between low EE and high EE
Wang et al. (2015)	China	CSS	152 dementia and caregivers	Outpatient clinics	Distress	NPI‐D	NPI‐subscale	Depressive symptoms in dementia were associated with caregiver distress (*r* = 0.25)
Wang et al. (2018)	China	CSS	210 dementia and caregivers	2 hospitals and 3 community settings	Burden, Anxiety, Depression, Positive aspect of caregiving Social support	CBI‐24 SAS SDS PAC Social Support Rating Scale (SSRS)	GDS‐30	Depressive symptoms in dementia were positively correlated with caregiver burden, caregiver anxiety (*r* = 0.739) and caregiver depressive symptoms (0.699). However it was negatively associated with the PAC scores (r = −0.279) PAC was a partial mediation of the depressive symptoms in dementia on caregiver anxiety, caregiver burden and caregiver depressive symptoms Social support moderates the relationship between patient depressive symptoms and caregiver burden, but it did not moderate the influence of depressive symptoms in dementia on caregiver anxiety and caregiver depressive symptoms
Watson et al. (2011)	USA	CSS	257 ad and caregivers	Nursing home	Burden	Burden interview BDI	CSDD	The patients' CSDD scores of less than 7 were predicted to caregiver burden
Wiglesworth et al. (2010)	USA	CSS	129 dementia and caregivers	community setting	elder mistreatment	Physical Assault and Psychological Aggression Scales from the Revised Conflict Tactics Scales (CTS2) Elder Abuse Instrument Safety of the Environment section of the Self‐Neglect Assessment Scale (SotE)	Structured Clinical Interview for the Diagnostic and Statistical Manual of Mental Disorders depression scale	There were no statistically significant differences in the mean scores of depressive symptoms in dementia to physical abuse with other mistreatment, neglect without physical abuse, psychological abuse only and the control group with no abuse or neglect
Wong and Zelman (2020)	China (HK)	CSS	89 dementia and caregivers	community setting	Burden Depression	ZBI‐22 CES‐D	NPI‐subscale	Depressive symptom in dementia was associated with caregiver depressive symptoms (*r* = 0.210) Depressive symptom in dementia was not associated with caregiver burden (*r* = 0)
Wright et al. (1998)	USA	LS	14 early‐phase AD and spouse	outpatient clinic	Spousal interaction (quality and quantity of interactions for cohesion, tension and affection; and commitment to the future of the relationship)	Dyadic Adjustment Rating	Short Zung Depression Rating Scale	Depressive symptom in dementia at baseline was associated with the quantity of tension (*r* = 0.533) and commitment to the future of the relationship at the 6‐month follow‐up (*r* = −0.506) Depressive symptom in dementia was not associated with other subscales of the spousal interaction (*r* = 0)
Xue et al. (2018)	China	CSS	168 ad (mild) and caregivers	2 hospitals and 3 community settings	Burden, Depression Anxiety positive aspects of caregiving	CBI SDS SAS PAC	GDS‐30	The interaction term of depressive symptoms of people with dementia and positive aspects of caregiving was negatively correlated (t = −3.09) Depressive symptoms were predicted to caregiver burden (*r* = 0.07), but not anxiety (*r* = 0) and depression (*r* = 0) The moderating positive aspects of caregiving on the association between depressive symptoms of dementia and caregiver burden, but it did not moderate the effect of depressive symptoms in dementia on caregiver anxiety and caregiver depressive symptoms
Yang et al. (2019)	China	CSS	157 dementia and caregivers	a local hospital in the rural	Burden; Finding positives in caregiving	The 12‐short version of ZBI, a subscale of a caregiver meaning scale	CSDD	The depressive symptoms of dementia were predicted to family caregiver burden (*r* = 0.12) The interaction term of depressive symptoms of dementia patients and finding positives in caregiving was negatively correlated After control confounder, the moderating role of finding positives in caregiving on the association between depressive symptoms of dementia patients and caregiver burden
Yilmaz et al. (2009)	Turkey	CSS	44 ad and caregivers	Home or at the psychiatry department	Burnout (MBI‐Emotional exhaustion, MBI‐Depersonalization, MBI‐Personal accomplishment)	MBI	GDS‐30	The MBI‐Depersonalization had a difference between non‐depressed patients and depressed patients The MBI‐Emotional exhaustion and MBI‐Personal had no difference between non‐depressed patients and depressed patients
Yoshino and Takechi (2023)	Japan	CSS	206 dementia and caregivers	Outpatient clinic	Burden	ZBI	GDS‐15	In multiple regression, depressive symptoms in people with dementia did not significantly predict caregiver burden (*r* = 0)
Zauszniewski and Burant (2020)	USA	CSS	138 dementia and caregivers	community setting	Resourcefulness Depressive symptoms	Resourcefulness Scale CES‐D	RMBPC‐subscale	Depressive symptom in dementia was associated with caregiver resourcefulness (*r* = −0.41) and caregiver depressive symptoms (*r* = 0.32)

Abbreviations: AD, Alzheimer's disease; ADRD, Alzheimer's disease and related dementia; BDI, Beck Depression Inventory; BRSDDEP, Behaviour Rating Scale for Dementia Depressive subscale; bvFTD, behavioural‐variant frontotemporal dementia; CBI, Caregiver Burden Inventory; CDS, Cornell Depression Scale; CES‐D, the Center for Epidemiologic Studies‐Depression scale; CSDD, Cornell Scale for Depression in Dementia; CSI, Caregiver Strain Index; CSS, cross‐sectional study; ECFR, Elder‐Caregiver‐Family Relationship Scale; EQ‐VAS, The Euro‐Qol Visual Analogue Scale; GAD‐7, Generalised Anxiety Disorder Scale‐7; GDS‐15, 15‐item Geriatric Depression Scale; GDS‐30, 30‐item Geriatric Depression Scale; GHQ‐12, 12‐item The General Health Questionnaire; GHQ‐30, 30‐item The General Health Questionnaire; GSS, Gilliard's Strain Scale; HADS, Hospital Anxiety and Depression Scale; HAM‐D, Hamilton Rating Scale for Depression; HDRS, Hamilton Depression Rating Scale; ISB, Inappropriate sexual behaviour; LS, longitudinal study; MBI, Maslach Burnout Inventory; NPI‐D, Neuropsychiatric Inventory Questionnaire distress score; PAC, positive aspects of caregiving; PHQ‐9, depression Patient Health Questionnaire scores; PMS, Pearlin Mastery Scale; PSS, Perceived Stress Scale; PwAD, people with Alzheimer's disease; PwD, people with dementia; QoL‐AD, The Quality of Life in Alzheimer's Disease Scale; RMBPC, Revised Memory and Behaviour Problems Checklist; SAS, Zung Anxiety Scale; ZBI, Zarit Burden Interview; ZCBS, Zarit Caregiver Scale; ZDS, Zung caregiver depression.

### 3.3 Summary of Methodological Quality

Table 2 presented the scores of the included studies using the Newcastle‐Ottawa Scale (NOS). Among the included cross‐sectional studies, the quality of most studies was moderate (*n* = 58). Others had low scores (*n* = 5) and high scores (*n* = 10). Among the included longitudinal studies, the quality of studies was low (*n* = 4). Others had moderate (*n* = 7) and high scores (*n* = 2). The quality score of the case–control study was six points.

**TABLE 2:** Quality assessment scores.

Author/year	Study design	Selection (maximum 5 stars)				Comparability (maximum 2 stars)	Outcome (maximum 3 stars)		Total scores
		1. Representativeness of the sample	2. Sample size	3. Non‐respondents	4. Ascertainment of the exposure (risk factor)	1. The subjects in different outcome groups are comparable, based on the study design or analysis. Confounding factors are controlled	1. Assessment of the outcome	2. Statistical test	
Allegri et al. (2006)	CSS	*			**		**	*	6
Ballard et al. (1997)	CSS			*	*		**	*	5
Bekhet and Garnier‐Villarreal (2020)	CSS	*	*		**	*	**	*	8
Braun et al. (2010)	CSS				**		**	*	5
Brækhus et al. (1998)	CSS	*		*	**		**	*	7
Brodaty and Luscombe (1998)	CSS	*			**		**	*	6
Bruvik et al. (2012)	CSS	*			**	*	**	*	7
Cheng, Ip, et al. (2013)	CSS	*			**	*	**	*	7
Cheng, Lam, et al. (2013)	CSS	*			**	*	**	*	7
Conde‐Sala et al. (2009)	CSS	*			**		**	*	6
da Rosa et al. (2016)	CSS	*			**	*	**	*	7
Deeken et al. (2018)	CSS	*		*	**	*	**	*	8
Delfino et al. (2021)	CSS	*			**		**	*	6
Donaldson et al. (1998)	CSS	*			**	*	**	*	7
García‐Martín et al. (2023)	CSS	*		*	**	**	**	*	9
Hanzevacki et al. (2023)	CSS	*			**	**	**	*	8
Harwood et al. (1998)	CSS	*			**		**	*	6
Hasegawa et al. (2014)	CSS	*			**	**	**	*	8
Hiyoshi‐Taniguchi et al. (2018)	CSS	*			**		**	*	6
Huang et al. (2012)	CSS	*			**		**	*	6
Huang et al. (2015)	CSS	*			**	**	**	*	8
Ikanga et al. (2023)	CSS	*			**		**	*	6
Ilik et al. (2020)	CSS	*		*	**		**	*	7
Iravani et al. (2022)	CSS	*		*	**		**	*	7
Kai et al. (2022)	CSS	*			**	*	**	*	7
Kaufer et al. (1998)	CSS	*			**		**	*	6
Khoo et al. (2013)	CSS	*			**	**	**	*	8
Lewis and Riley (2021)	CSS	*	*		**		**	*	7
Lima‐Silva et al. (2015)	CSS	*			**	*	**	*	7
Linton (2020)	CSS (Data is presented from baseline)	*	*		**		**	*	7
Lou et al. (2015)	CSS	*			**		**	*	6
Lu et al. (2022)	CSS	*	*	*	**	**	**	*	10
Magai and Cohen (1998)	CSS	*			**		**	*	6
Matsumoto et al. (2007)	CSS	*		*	**		**	*	6
Miller et al. (2019)	CSS	*			**	**	**	*	8
Morgan et al. (2013)	CSS (Data is presented from baseline survey)				**		**	*	5
Nogales‐González et al. (2015)	CSS	*		*	**	**	**	*	9
Nogueira et al. (2017)	CSS	*			**	**	**	*	8
Nogueira et al. (2015)	CSS	*			**	**	**	*	8
K. Ornstein et al. (2013)	CSS (Data is presented from baseline survey)	*	*	*	**	**	**	*	10
Ottoboni et al. (2019)	CSS (Data is presented from baseline survey)	*			**		**	*	6
Pang et al. (2002)	CSS	*			**	**	**	*	8
Papastavrou et al. (2007)	CSS	*		*	**	**	**	*	9
Parrotta et al. (2020)	CSS (Data is presented from the baseline survey)	*			**	**	**	*	8
Pöysti et al. (2012)	CSS	*			**	**	**	*	8
Raggi et al. (2017)	CSS	*			**	**	**	*	8
Regier et al. (2020)	CSS (baseline data from a longitudinal behavioural intervention study)	*			**	*	**	*	7
Robinson et al. (2001)	CSS	*	*		**		**	*	7
Rosa et al. (2020)	CSS	*			**	**	**	*	8
Sakar et al. (2019)	CSS	*			**	**	**	*	8
Schumann et al. (2019)	CSS	*			**		**	*	6
Shi and Scott (2023)	CSS	*	*		**	**	**	*	9
Shin et al. (2005)	CSS	*			**		**	*	6
Simpson and Carter (2013)	CSS	*		*	**	**	**	*	9
Sousa et al. (2016)	CSS	*			**	*	**	*	7
Springate and Tremont (2014)	CSS	*			**		**	*	6
Sutcliffe et al. (2017)	CSS	*	*		**	**	**	*	9
Tayeb et al. (2023)	CSS	*	*		**		**	*	7
Toda et al. (2018)	CSS	*		*	**	**	**	*	9
Torrisi et al. (2017)	CSS	*			**		**	*	6
Truzzi et al. (2012)	CSS	*			**	**	**	*	8
Tsai et al. (2022)	CSS	*			**	**	**	*	8
Victoroff et al. (1998)	CSS	*			**		**	*	6
Wagner et al. (1997)	CSS	*		*	**		**	*	7
Wang et al. (2015)	CSS	*	*		**	**	**	*	9
Wang et al. (2018)	CSS	*		*	**	**	**	*	9
Watson et al. (2001)	CSS	*		*	**		**	*	7
Wiglesworth et al. (2010)	CSS	*			**	*	**	*	7
Wong and Zelman (2020)	CSS	*			**		**	*	6
Xue et al. (2018)	CSS	*			**	**	**	*	8
Yang et al. (2019)	CSS	*			**	**	**	*	8
Yilmaz et al. (2009)	CSS	*			**	**	**	*	8
Yoshino and Takechi (2023)	CSS	*			**		**	*	6
Zauszniewski and Burant (2020)	CSS (baseline data from a clinical trial)	*	*		**	**	**	*	9

Author/year	Study design	Selection				Comparability	Outcome			Total scores
		1. Representativeness of the exposed cohort	2. Selection of the non‐exposed cohort	3. Selection of Controls	4. Definition of Controls	5. Comparability of cases and controls on the basis of the design or analysis	6. Assessment of outcome	7. Was follow‐up long enough for outcomes to occur	8. Adequacy of follow‐up of cohort	
Burgener and Dickerson‐Putman (1999)	LS	*		*			*	*	*	5
Burgener and Twigg (2002)	LS			*		*	*	*	*	5
Cai et al. (2023)	LS	*		*		*	*	*		5
Caron et al. (1999)	LS	*		*			*	*		4
Gellert et al. (2018)	LS			*		**	*	*		5
Hallikainen et al. (2018)	LS	*		*		**	*	*		6
Lethin et al. (2020)	Cohort study	*		*		*	*		*	5
Neundorfer et al. (2001)	LS			*			*	*		4
K. A. Ornstein et al. (2013)	LS	*		*		**	*	*	*	7
Porta‐Etessam et al. (2011)	Cohort study	*	*	*	*	**	*	*		8
Safavi et al. (2023)	LS	*		*		*	*	*		5
Safavi et al. (2019)	LS			*		*	*	*		4
Wright et al. (1998)	LS			*			*	*		3

Author/year	Study design	Selection				Comparability	Outcome			Total scores
		1. Is the case definition adequate?	2. Representativeness of the cases	3. Selection of Controls	4. Definition of Controls	5. Comparability of cases and controls on the basis of the design or analysis	6. Ascertainment of exposure	7. Same method of ascertainment for cases and controls	8. Non‐Response rate	
Nogueira et al. (2017)	Case–control study	*	*	*	*		*	*		6

*Note:* In the cross‐sectional study, a maximum of two stars can be given for ascertainment of the exposure (risk factor), Comparability and assessment of the outcome. Others can be given one star for each fulfilled item; In the longitudinal study and case‐control study, a study can be given one star for each fulfilled item in the Selection and Outcome. A maximum of two stars can be given for Comparability.

Abbreviations: CSS, cross‐sectional study; LS, longitudinal study.

### 3.4 Data Synthesis

#### 3.4.1 All Primary Synthesis

**FIGURE 2:**
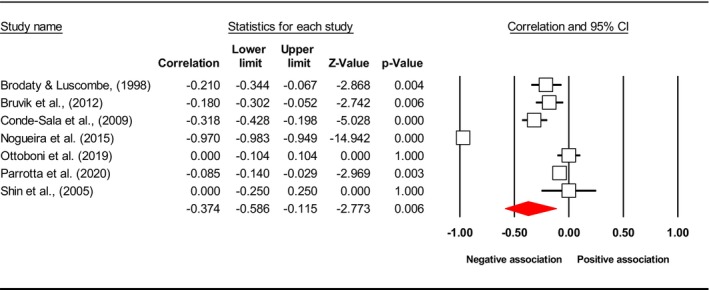
Forest plot of the relationship between caregiver QoL level and depressive symptoms in people with dementia. The common metric for the effect size in each study is the Fisher's *r*‐to‐*z* transformation of the correlation statistics. The white boxes represent the Fisher's *Z* estimates for individual studies, with the horizontal lines on either side of each box represent the 95% confidence intervals (CIs) for the estimated value of that study. The red diamond represents the overall mean Fisher's *Z*, with the middle of the diamond indicating the pooled estimate and the left and right extremes representing the 95% CI. The *p*‐value indicates the statistical significance of the Fisher's *Z* estimate for each study. A *p*‐value < 0.05 suggests that the effect size is statistically significant and unlikely to be due to random chance, while a *p*‐value 0.05 suggests that the effect size is not statistically significant.

**FIGURE 3:**
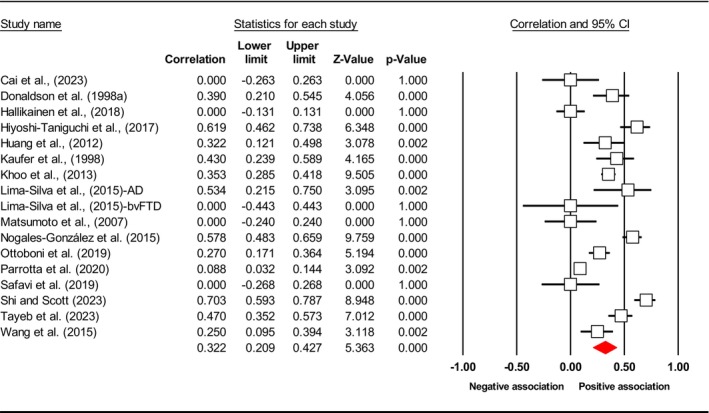
Forest plot of the relationship between caregiver distress level and depressive symptoms in people with dementia. The common metric for the effect size in each study is the Fisher's *r*‐to‐*z* transformation of the correlation statistics. The white boxes represent the Fisher's *Z* estimates for individual studies, with the horizontal lines on either side of each box represent the 95% confidence intervals (CIs) for the estimated value of that study. The red diamond represents the overall mean Fisher's *Z*, with the middle of the diamond indicating the pooled estimate and the left and right extremes representing the 95% CI. The *p*‐value indicates the statistical significance of the Fisher's *Z* estimate for each study. A *p*‐value < 0.05 suggests that the effect size is statistically significant and unlikely to be due to random chance, while a *p*‐value 0.05 suggests that the effect size is not statistically significant.

**FIGURE 4:**
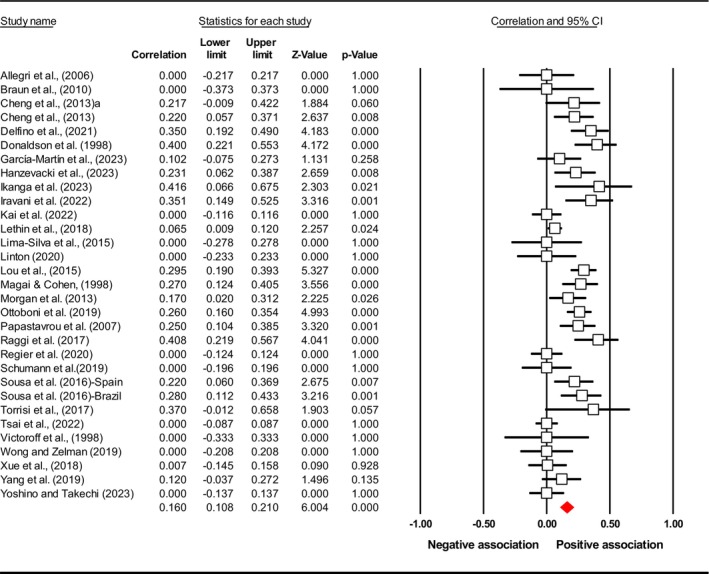
Forest plot of the relationship between caregiver burden level and depressive symptoms in people with dementia. The common metric for the effect size in each study is the Fisher's *r*‐to‐*z* transformation of the correlation statistics. The white boxes represent the Fisher's *Z* estimates for individual studies, with the horizontal lines on either side of each box represent the 95% confidence intervals (CIs) for the estimated value of that study. The red diamond represents the overall mean Fisher's *Z*, with the middle of the diamond indicating the pooled estimate and the left and right extremes representing the 95% CI. The *p*‐value indicates the statistical significance of the Fisher's *Z* estimate for each study. A *p*‐value < 0.05 suggests that the effect size is statistically significant and unlikely to be due to random chance, while a *p*‐value 0.05 suggests that the effect size is not statistically significant.

**FIGURE 5:**
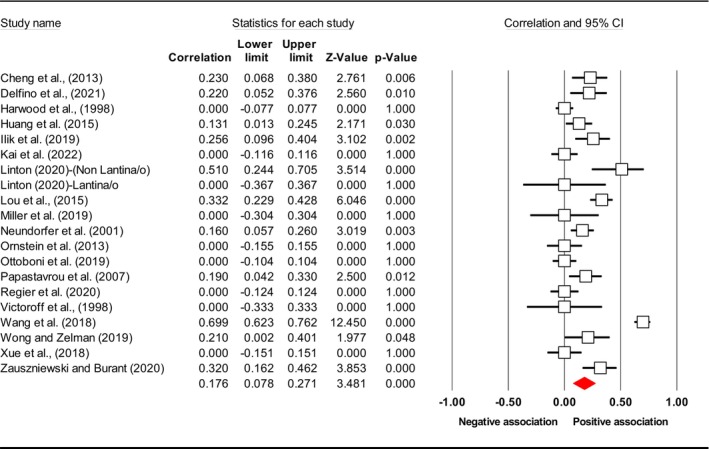
Forest plot of the relationship between caregiver depression level and depressive symptoms in people with dementia. The common metric for the effect size in each study is the Fisher's *r*‐to‐*z* transformation of the correlation statistics. The white boxes represent the Fisher's *Z* estimates for individual studies, with the horizontal lines on either side of each box represent the 95% confidence intervals (CIs) for the estimated value of that study. The red diamond represents the overall mean Fisher's *Z*, with the middle of the diamond indicating the pooled estimate and the left and right extremes representing the 95% CI. The *p*‐value indicates the statistical significance of the Fisher's *Z* estimate for each study. A *p*‐value < 0.05 suggests that the effect size is statistically significant and unlikely to be due to random chance, while a *p*‐value 0.05 suggests that the effect size is not statistically significant.

**FIGURE 6:**
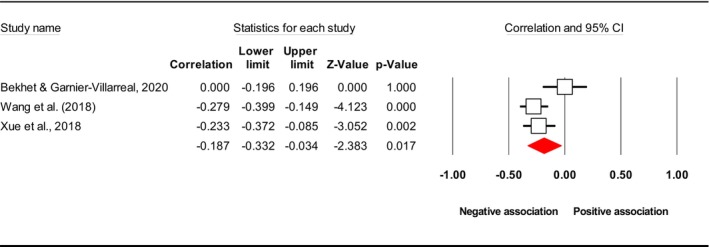
Forest plot of the relationship between the level of caregiver PAC and depressive symptoms in people with dementia. The common metric for the effect size in each study is the Fisher's *r*‐to‐*z* transformation of the correlation statistics. The white boxes represent the Fisher's *Z* estimates for individual studies, with the horizontal lines on either side of each box represent the 95% confidence intervals (CIs) for the estimated value of that study. The red diamond represents the overall mean Fisher's *Z*, with the middle of the diamond indicating the pooled estimate and the left and right extremes representing the 95% CI. The *p*‐value indicates the statistical significance of the Fisher's *Z* estimate for each study. A *p*‐value < 0.05 suggests that the effect size is statistically significant and unlikely to be due to random chance, while a *p*‐value 0.05 suggests that the effect size is not statistically significant.

**FIGURE 7:**
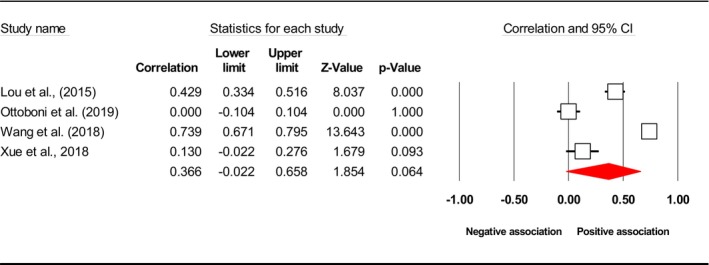
Forest plot of the relationship between caregiver anxiety level and depressive symptoms in people with dementia. The common metric for the effect size in each study is the Fisher's *r*‐to‐*z* transformation of the correlation statistics. The white boxes represent the Fisher's *Z* estimates for individual studies, with the horizontal lines on either side of each box represent the 95% confidence intervals (CIs) for the estimated value of that study. The red diamond represents the overall mean Fisher's *Z*, with the middle of the diamond indicating the pooled estimate and the left and right extremes representing the 95% CI. The *p*‐value indicates the statistical significance of the Fisher's *Z* estimate for each study. A *p*‐value < 0.05 suggests that the effect size is statistically significant and unlikely to be due to random chance, while a *p*‐value 0.05 suggests that the effect size is not statistically significant.

**FIGURE 8:**
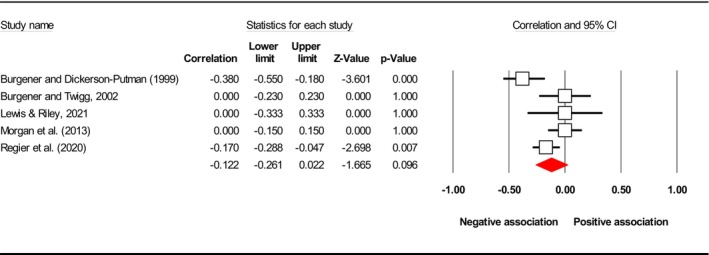
Forest plot of the relationship between the level of quality of dyadic relationship and depressive symptoms in people with dementia. The common metric for the effect size in each study is the Fisher's *r*‐to‐*z* transformation of the correlation statistics. The white boxes represent the Fisher's *Z* estimates for individual studies, with the horizontal lines on either side of each box represent the 95% confidence intervals (CIs) for the estimated value of that study. The red diamond represents the overall mean Fisher's *Z*, with the middle of the diamond indicating the pooled estimate and the left and right extremes representing the 95% CI. The *p*‐value indicates the statistical significance of the Fisher's *Z* estimate for each study. A *p*‐value < 0.05 suggests that the effect size is statistically significant and unlikely to be due to random chance, while a *p*‐value 0.05 suggests that the effect size is not statistically significant.

Table 3 showed the main results of the meta‐analysis for each caregiver's psychosocial factors, including the number of studies (*n*), pooled effect size (*r*), 95% confidence intervals, degree of heterogeneity (*I* ^2^), publication bias (*p* using Egger's test) and included study. Subsequently, the final meta‐analysis explored seven distinct exposures (caregiver QoL (Figure 2), caregiver distress (Figure 3), caregiver burden (Figure 4), caregiver depression (Figure 5), PAC (Figure 6), caregiver anxiety (Figure 7) and dyadic quality of relationship (Figure 8)).

**TABLE 3:** Pooled effect size estimates, heterogeneity and publication bias.

Caregiver psychosocial factors	No of studies	Effect size *r* [95% CI]	*I* ^2^ (%)	Egger's test, *p*	Included study
QoL	7	−0.374 [−0.586, −0.115]	97.211	NA	Brodaty and Luscombe (1998), Bruvik et al. (2012), Conde‐Sala et al. (2009), Nogueira et al. (2015), Ottoboni et al. (2019), Parrotta et al. (2020), Shin et al. (2005)
Distress	16	0.322 [0.209, 0.427]	91.243	0.212	Cai et al. (2023), Donaldson et al. (1998), Hallikainen et al. (2018), Hiyoshi‐Taniguchi et al. (2018), Huang et al. (2012), Kaufer et al. (1998), Khoo et al. (2013), Lima‐Silva et al. (2015), Matsumoto et al. (2007), Nogales‐González et al. (2015), Ottoboni et al. (2019), Parrotta et al. (2020), Safavi et al. (2019), Shi and Scott (2023), Tayeb et al. (2023), Wang et al. (2015)
PAC	3	−0.187 [−0.332, −0.034]	64.399	NA	Bekhet and Garnier‐Villarreal (2020), Wang et al. (2018), Xue et al. (2018)
Depression	19	0.176 [0.078, 0.271]	89.255	0.381	Cheng, Lam, et al. (2013), Delfino et al. (2021), Harwood et al. (1998), Huang et al. (2015), Ilik et al. (2020), Kai et al. (2022), Linton (2020), Lou et al. (2015), Miller et al. (2019), Neundorfer et al. (2001), Ornstein et al. (2013), Ottoboni et al. (2019), Papastavrou et al. (2007), Regier et al. (2020), Victoroff et al. (1998), Wang et al. (2018), Wong and Zelman (2020), Xue et al. (2018), Zauszniewski and Burant (2020)
Burden	32	0.160 [0.108, 0.210]	69.509	0.081	Allegri et al. (2006), Braun et al. (2010), Cheng, Ip, et al. (2013) , Cheng, Lam, et al. (2013), Delfino et al. (2021), Donaldson et al. (1998), García‐Martín et al. (2023), Hanzevacki et al. (2023), Ikanga et al. (2023), Iravani et al. (2022), Kai et al. (2022), Lethin et al. (2020), Lima‐Silva et al. (2015), Linton (2020), Lou et al. (2015), Magai and Cohen (1998), Morgan et al. (2013), Ottoboni et al. (2019), Papastavrou et al. (2007), Parrotta et al. (2020), Pöysti et al. (2012), Raggi et al. (2017), Regier et al. (2020), Schumann et al. (2019), Sousa et al. (2016), Torrisi et al. (2017), Tsai et al. (2022), Victoroff et al. (1998), Wong and Zelman (2020), Xue et al. (2018), Yang et al. (2019), Yoshino and Takechi (2023)
quality of relationship	5	−0.122 [−0.261, 0.022]	63.252	NA	Burgener and Dickerson‐Putman (1999), Burgener and Twigg (2002), Lewis and Riley (2021), Morgan et al. (2013), Regier et al. (2020)
Anxiety	4	0.336 [−0.022, 0.658]	97.67	NA	Lou et al. (2015), Ottoboni et al. (2019), Wang et al. (2018), Xue et al. (2018)

*Note:* If the factors included in the meta‐analysis were less than ten articles, then the Egger's test would not be performed.

Abbreviation: NA, not applicable.

Caregiver distress and caregiver QoL demonstrated effect sizes ranging from medium to large (specifically, in the range of 0.30 to 0.50). Caregiver QoL had the most significant effect size (*r* = −0.37; 95% CI, −0.59 to −0.12), but only seven studies were in this review. Caregiver distress had the second largest effect size (*r* = 0.32; 95% CI, 0.21, 0.43), and 16 studies were included in this meta‐analysis.

Three factors had small to medium effect sizes (i.e., between 0.10 and 0.30). They were caregiver burden, caregiver depression and PAC. Caregiver burden (*n* = 32) was examined in the greatest number of studies, and this factor had an effect size of 0.16 regarding depressive symptoms in dementia. Caregiver depression (*n* = 19) had an effect size of 0.18, and PAC (*n* = 3) had an effect size of −0.19. Another two factors, including the quality of dyadic relationships and caregiver anxiety, were not significant.

Caregiver distress and caregiver QoL had a high heterogeneity (*I* ^2^ > 91%). However, the heterogeneity for caregiver burden was *I* ^2^ = 69.51%, and for depression, it was *I* ^2^ = 89.26%. The results indicated no significant publication bias among these seven caregiver psychosocial factors, with all Egger's *p*‐values more than 0.05.

#### 3.4.2 Meta‐Regression

In the meta‐regression analysis, the age of dementia patients, caregiver age and the percentage of female caregivers emerged as potential confounding factors influencing the relationships between caregiver burden, distress, depression and depressive symptoms in PwD. For caregiver distress, we also found a moderating effect on the percentage of females (*β* = −0.02, *p* = 0.012). As the percentage of females increased, the value of the correlation coefficient increased. Hence, for female caregivers, caregiver distress had a stronger association with depressive symptoms in PwD. No statistically significant associations were observed in other examined moderators.

## 4 Discussion

This meta‐analysis was the first to explore the association between caregiver QoL, caregiver distress, caregiver burden, caregiver depression, PAC, caregiver anxiety, quality of dyadic relationships and depressive symptoms in PwD. Caregiver burden, distress and depression were positively correlated with depressive symptoms in PwD. Better QoL and PAC were negatively associated with depressive symptoms in PwD. There was no relationship between caregiver anxiety, quality of dyadic relationships and depressive symptoms in PwD. Identifying caregiver psychosocial factors associated with depressive symptoms in PwD was crucial for shaping policies, treatments and supportive measures that maintain the psychosocial health of dyads.

### 4.1 Moderately Associated Caregiver Psychosocial Factors

Of all the caregiver psychosocial factors in this review, caregiver QoL and distress were paramount predictors of depressive symptoms in PwD, with caregiver QoL exhibiting the strongest correlation (*r* = −0.374) and caregiver distress following closely (*r* = 0.322). Caregiver distress and QoL were interdependent, with distress reducing QoL and low QoL enlarging distress (Pearlin et al. 1990). This mutual relationship significantly impacted depressive symptoms in PwD (Monin et al. 2021). Factors like inadequate social support, poor coping mechanisms, and other behavioural challenges in PwD can simultaneously influence caregiver distress and QoL, creating a compounded effect on depressive symptoms in PwD (Pearlin et al. 1990). These multifactorial and interconnected influences inherently result in moderate correlation coefficients, reflecting the complexity and interplay of psychosocial factors within caregiving dynamics.

The QoL of caregivers and PwD was interdependent, with changes in one member's QoL often reflecting or influencing the others (Wang, Huang, et al. 2024). Dyadic relationship strain can negatively affect the QoL of caregivers and PwD (Miller et al. 2019). This dynamic can be further exacerbated by differing perceptions of the relationship between caregivers and PwD (Moon et al. 2017). Inconsistent expectations or understanding of care needs can lead to frustration, and reducing the dyadic QoL.

The meta‐regression analysis identified the percentage of female caregivers as a significant moderator influencing the relationship between caregiver distress and depressive symptoms in PwD. Female caregivers may rely more heavily on emotion‐focused coping strategies, which could exacerbate distress and have a greater impact on depressive symptoms in PwD (Sharma et al. 2016). Women may have higher levels of emotional involvement in caregiving (Sharma et al. 2016), which could amplify the bidirectional emotional connection between caregiver distress and depressive symptoms in PwD. Female caregivers often need to balance caregiving with other roles, such as employment and family responsibilities (Mussida and Patimo 2021), leading to increased strain and distress. This multifaceted domain could intensify the impact of caregiver distress on PwD depressive symptoms.

Stress management techniques, like mindfulness and exercise can improve caregiver QoL, distress and depressive symptoms in PwD (Cho et al. 2023; Sun et al. 2022). Interventions that target caregiver–patient dyads might optimise caregiver psychosocial factors and ultimately help address depressive symptoms in PwD. Researchers should conduct more longitudinal studies to assess how improvements in the psychosocial health of caregivers may affect depressive symptoms in dementia patients over the long term, leading to insights into the development of interventions. Besides, research should aim to develop and evaluate personalised interventions to meet specific dyadic needs and identify the most effective approaches based on individual and relational factors.

### 4.2 Lowly Associated Caregiver Psychosocial Factors

Compared with caregiver distress and QoL, caregiver depression, burden and PAC were weaker associated factors of depressive symptoms in PwD, yielding a small to moderate effect size. Despite the small effect sizes observed, the relationship between caregiver burden, caregiver depression and positive aspects of caretaking has been elucidated in the literature. Long‐term caregiver burden, exacerbated by inadequate support and constant stress, often leads to caregiver depression (Liu et al. 2020). Caregiver depression can harm the health of caregivers, and the quality of care provided, extending the cycle of worsening care outcomes (Liu et al. 2020; Proulx et al. 2017). However, PAC, such as emotional satisfaction, strengthened relationships and a sense of accomplishment, may buffer these adverse effects and may prevent the onset of caregiver depression (Proulx and Aldwin 2016). If the negative effects of caregiving overwhelm these positive experiences, caregivers may experience increasingly severe depression. Within the Systemic‐Transactional Model of dyadic coping, stress perceived by one partner is conveyed to the other verbally or non‐verbally, leading to dyadic coping (Bodenmann et al. 2019). Moreover, recent research has shown that individuals subconsciously convey emotions difficult to conceal through various signals, making facial and verbal expressions (Low et al. 2020; Zhang et al. 2020), emphasising the complexity of emotional communication within dyads. This phenomenon emphasised the interdependence within the caregiver–patient dyads. Hence, it is necessary to enhance the understanding of the complex dynamics involved in caregiving for caregiver depression and support more effective interventions that improve outcomes for depressive symptoms in PwD.

### 4.3 Not Associated Caregiver Psychosocial Factors

Interestingly, our analysis revealed no significant relationship between the dyadic relationship quality or caregiver anxiety and depressive symptoms in PwD. However, in Coyne's interpersonal process to depression model, the dyadic relationship quality was a critical factor in influencing depression (Coyne et al. 1990). A potential explanation for this apparent discrepancy might be due to the evaluation of the dyadic relationship quality between the caregiver and PwD, which should not solely focus on the caregiver's perspective (Burgener and Twigg 2002; Burgener and Dickerson‐Putman 1999). A systematic review explored the quality of dyadic relationships between caregivers and PwD, specifically assessing the quality of relationships from dyadic perspectives, which had different outcomes (Edwards et al. 2018). The variability in results suggested that the adopted perspectives of either caregivers or PwD may significantly influence relationship assessments. Hence, it is crucial to also consider evaluating the relationship quality from the perspective of caregivers and PwD. Meanwhile, it demonstrated a significant correlation between caregiver anxiety and depressive symptoms in PwD (Farina et al. 2017). However, contrary to what might be expected, our meta‐analysis found no such link between caregiver anxiety and depressive symptoms in PwD. Existing studies in parent–child relationships have demonstrated the transmissibility of anxiety through dyadic social dynamics (Perlman et al. 2022). This insight was particularly relevant in dementia caregiving, where both parties often employ emotion‐focused coping strategies (Colclough et al. 2023). These strategies required communicating stress or anxiety signals to each other and then working together to manage these emotional states collaboratively (Wang, Huang, et al. 2024). Therefore, it is necessary to clarify the mutual influence of emotions within dyads and to explore the mechanisms underlying their emotional interplay, particularly in the context of caregiver anxiety and depressive symptoms in PwD.

### 4.4 Interactions Among Psychosocial Factors and External Influences

The Stress Process Model (SPM) provided a theoretical framework to examine the interconnectedness of stressors, resources and psychological well‐being in caregiving (Pearlin et al. 1990). Although distinct, the seven psychosocial factors explored in this review often overlap due to the presence of common underlying factors, such as stress, coping mechanisms and social support (Wang et al. 2023).

Caregiver burden and distress were often interlinked, as both are responses to the demands and challenges of caregiving. Burden encompassed the physical, emotional and financial strain experienced by caregivers (Liu et al. 2020). Caregiver distress primarily refers to the psychological and emotional impacts, such as anxiety and depression (Lee et al. 2024). Caregiver burden and psychological distress were mutually influential, with each exacerbating the other and collectively contributing to a decline in QoL (Cheng et al. 2022). Psychological well‐being was characterised by the presence of positive emotions (high self‐esteem) and the absence of negative emotions (anxiety or depression) (Stoll and Pollastri 2023). It played a critical role in the caregiving context, as mental health challenges like anxiety and depression are closely linked to caregiver burden and distress, significantly influencing overall caregiver QoL. The targeted enhancement of psychological well‐being through interventions such as psychoeducation, leisure and physical activities, counselling, cognitive behavioural approaches and befriending or peer support can alleviate caregiver burden and distress while improving QoL (Wiegelmann et al. 2021).

The availability of social support was a critical mediator in caregiving (Wang et al. 2018). It can reduce perceived burden and distress while enhancing the caregiver's QoL and depressive symptoms in PwD (Wang et al. 2018). Programs like the Stress Process Model‐Based Program in Dementia Caregiving emphasised the role of support networks in caregiving (Wang et al. 2023). Effective coping strategies, whether problem‐based or emotion‐based, played a pivotal role in managing caregiving challenges (Bodenmann et al. 2019).

### 4.5 Limitation

It should be considered that there were several limitations of this systematic review. At first, different tools were used to assess depressive symptoms in PwD, and participant group heterogeneity was clear. Second, some caregiver factors were less reported in the previous study, making it impossible for a meta‐analysis. Third, most of the research used cross‐sectional designs, which naturally restrict the ability to demonstrate causal mechanisms. Furthermore, the significant heterogeneity observed among the included studies suggests the influence of unexplored effect modifiers. Finally, the review exclusively encompassed articles published in English, potentially resulting in incomplete retrieval of research due to language restrictions.

### 4.6 Implications for Future Research and Practice

This meta‐analysis highlighted the complex interplay between psychosocial factors among caregivers and depressive symptoms in PwD, advocating a clinical approach that went beyond focusing solely on either side. Nurses and healthcare professionals need to take a comprehensive approach, including dyads. Dyadic intervention plans that consider the dyadic needs emphasise that healthcare professionals must have the specialised knowledge and skills to implement effective interventions proactively. These interventions are essential to ensure that caregivers and PwD receive comprehensive support to meet the psychosocial needs of caregivers and improve depressive symptoms in PwD. In addition, emotional communication skills highlight healthcare professional skills that promote open expression and management of psychosocial factors in caregivers and depressive symptoms in patients with dementia. This approach facilitates emotional regulation and enhances the support network for dyads involved in the dementia care process.

In clinical practice, the findings highlight the transformation towards dyadic care models that simultaneously address the needs of caregivers and PwD. Nurses play a crucial role in this progress, as they are uniquely positioned to deliver comprehensive care and support. Nursing interventions should be tailored to manage caregivers' stress and offer emotional support programmes. Routine nursing assessments should incorporate comprehensive psychosocial evaluations to identify the needs of caregivers and PwD. By addressing these needs proactively, healthcare professionals can mitigate depressive symptoms in PwD and improve caregiver psychological well‐being, ultimately improving outcomes for dyads.

## 5 Conclusion

Effective dementia care requires addressing the needs of both patients and caregivers. Future research should explore longitudinal dynamics to clarify the causal pathways between caregiver psychosocial factors and depressive symptoms in PwD. We also recommend tailored interventions that consider the diverse experiences and needs of dyads. Given the link between caregiver psychosocial factors and depressive symptoms in people with dementia, policymakers should prioritise funding for caregiver support services and consider integrating dyadic programmes into dementia care frameworks to ensure sustainable and equitable care.

## Author Contributions

**Wenjing Ning:** conceptualization, methodology, data collection and writing original draft. **Shanshan Wang:** writing, review, editing and project administration. **Yudi Xu:** data collection, analysis and interpretation of results. **Daphne Cheung:** supervision, writing, review, editing, project administration.

## Conflicts of Interest

The authors declare no conflicts of interest.

## Data Availability

Data sharing not applicable.
